# Editorial: Identification, development and use of rootstocks to improve pest and disease resistance of vegetable crops

**DOI:** 10.3389/fpls.2023.1320828

**Published:** 2023-12-12

**Authors:** Judy A. Thies, Dilip R. Panthee

**Affiliations:** ^1^ U.S. Vegetable Laboratory, Agricultural Research Service, U.S. Department of Agriculture, Charleston, SC, United States; ^2^ Department of Horticultural Science, College of Agriculture and Life Sciences, North Carolina State University, Mills River, NC, United States

**Keywords:** disease resistant rootstocks, grafting fruit tree crops, grafting vegetable crops, nematode resistant rootstocks, breeding for resistance, scion-rootstock interactions

Grafting susceptible crop plants on disease and pest-resistant rootstocks is a valuable management practice for reducing damage caused by plant-parasitic nematodes and plant pathogens in vegetable and fruit tree crops worldwide. Resistant rootstocks in the Solanaceae are used extensively for grafting tomato, eggplant, and pepper crops to manage a wide range of diseases and nematodes. Resistant Cucurbitaceous rootstocks have been developed for grafting watermelon, cucumbers, luffa, and melon. Scions of several fruit tree species (including susceptible citrus, apple, and olive) are grafted on resistant rootstocks, especially for managing soil-borne diseases and plant-parasitic nematodes. Grafting is a widely used alternative to soil fumigation and other pesticides for controlling soil-borne diseases and nematode pests. Rootstocks of several crops have been developed with specific resistance(s) to soil-borne diseases and plant-parasitic nematodes, including Verticillium wilt, Fusarium wilt, Fusarium crown and root rots, Southern blight, bacterial wilt, Huanlongbing (HLB), Phytophthora root rot, citrus tristeza virus, citrus canker (*Xanthomonas axonopodis*), *Meloidogyne incognita, M. arenaria, M. javanica*, and apple replant disease (a complex of *Phytophthora, Pythium, Cylindrocarpon*, and *Rhizoctonia* spp. interacting with the root-lesion nematode, *Pratylenchus penetrans*).

Southern root-knot nematode (*M. incognita*) susceptible tomato grafted on nematode-resistant rootstocks reduced root galling and increased yields (Kunwar et al., 2015; Frey et al., 2020). Grafting tomatoes susceptible to bacterial wilt (*Ralstonia solanacearum*) on resistant rootstocks had 88% to 125% higher fruit yields than non-grafted tomatoes (Suchoff et al., 2019).

*Meloidogyne incognita* causes root galling, plant stunting, and reduced fruit yield in watermelon. Wild watermelon rootstocks resistant to Southern root-knot nematode have been developed for use in grafted watermelon (Thies et al., 2015a). Watermelon grafted on nematode resistant rootstocks have reduced root galling and nematode root numbers, healthy plant growth, and significantly greater fruit yields than non-grafted watermelon (Thies et al., 2015b; García-Mendívil and Sorribas, 2021).

Citrus huanglongbing (HLB) is a devastating citrus disease associated with *Candidatus* Liberbacter asiaticus (CLas) and vectored to citrus by the Asian citrus psyllid (ACP). HLB causes phloem collapse and metabolic disorders, resulting in increased starch in leaves and depletion in roots. The scion affects ACP infestation and Clas colonization during infectious stages more than the rootstock (Tardivo et al., 2023). Differences in stress tolerance and efficiency of water and nutrient uptake may impact rootstock tolerance of grafted trees during later stages of HLB.

Fusarium wilt is an important disease of cucurbit crops (luffa, cucumbers, squash, watermelon) which causes severe yield losses. In Taiwan, Fusarium wilt has caused major losses in luffa production for several years (Lin and Su, 2001). The cultivar ‘White Loofah’ suffers up to 95% losses due to Fusarium wilt. Cultivated species of luffa (*Luffa aegyptiaca* and *L. acutangular*) are very susceptible to aggressive isolates of *Fusarium oxysporum* which cause severe disease and yield losses. Luffa rootstocks are used for cucurbit crops, but there is scant information on the resistance of these rootstocks to soilborne diseases.

Bowman et al. evaluated 46 new hybrid citrus rootstocks for genetic modulation of Valencia sweet orange in the field, under an huanglongbing (HLB) endemic environment. They found that a particular gene is linked to tolerance of HLB through the rootstock. By combining pedigree-based genetic information with quantitative phenotypic data, it would be easier to identify and select rootstocks with superior trait combinations needed for rootstock development using marker-assisted-selection, enabling rapid selection of successive-generation rootstocks with the desirable trait combinations for development of marketable rootstocks. Hybrids US-1649, US-1688, US-1709, and US-2338 were the most valuable new rootstocks, and may be released for commercial use pending future evaluations.

Bindal et al. evaluated 63 Luffa accessions from the World Vegetable Center genebank for resistance to an aggressive luffa isolate of *Fusarium oxysporum* f. FoCu-1 (Fsp-66). Fourteen of 63 accessions were highly resistant, and in further tests, 11 were highly resistant to Fsp-66 (luffa isolate), and 13 were highly resistant to both FoCu-1 (cucumber isolate) and FoM-6 (bitter gourd isolate). This is the first report of resistance to *F. oxysporum* in luffa. These accessions will be valuable in breeding luffa cultivars and rootstocks with Fusarium wilt resistance.

Fullana et al. conducted a rotation of four vegetable crops (tomato, melon, pepper, watermelon) grafted on rootstocks resistant to *Meloidogyne incognita* or not grafted. The scion/rootstock combinations were ‘Durinta’ tomato (*Solanum lycopersicum* L.)/’Brigeor’ tomato, ‘Paloma’ melon (*Cucumis melo* L.)/*Cucumis metulif*er accession BGV11135, ‘Tinsena’pepper (*Capscium annuum* L.)/’Oscos’pepper, and ‘Sugar Baby’ watermelon (*Citrullus lanatus*)/*Citrullus amarus* accession BGV5167 (COMAV-UPV), followed by susceptible (‘Durinta’) or resistant (‘Caramba’) tomato at the end of the rotation. The grafted and non-grafted treatments were grown in plots infested with an avirulent (AVI) or partially virulent (VI) population of *M. incognita*. Initial populations (Pi) of *M. incognita* in AVI and VI did not differ between grafted and non-grafted plants. At the end of the rotation, the final population (Pf) of the AVI subpopulation was 1.2 times >Pi for non-grafted plants and 0.06 times >Pi for grafted plants. The cumulative yield of all grafted crops was 1.83 times >non-grafted crops. Resistance of all four rootstocks was effective against both AVI and VI *M. incognita* isolates by reducing the Pf of both isolates and significantly increasing yields in grafted versus non-grafted crops.

Pablo Díaz-Rueda et al. demonstrated that Verticillium wilt (*Verticillium dahliae* Kleb.) in susceptible olive cultivar scions grafted on avoidant/resistant olive rootstocks, which limit fungal growth and rootstock infection, was more effective in controlling Verticillium wilt than grafting on tolerant rootstocks. It is important to consider the scion/rootstock interaction as it can affect rootstock performance. For example, when a susceptible scion is grafted on a rootstock with low susceptibility, the rootstock susceptibility may be increased. Therefore, it is important to evaluate each scion/rootstock combination and not rely on rootstock performance alone since there may be unexpected scion/rootstock interactions.

The articles published in this Research Topic focus on the development of resistant rootstocks through traditional breeding. Novel biotechnology approaches such as RNAi gene silencing and genome editing could also be very useful in developing new improved pest resistant rootstocks for annual and perennial horticultural crops. RNAi gene silencing has been utilized to develop tomato resistance to both *Meloidogyne incognita* and tomato leaf curl virus (Koulagi et al., 2020). The citrus canker susceptibility gene *CSLOB1* was modified using genome editing (CRISPR biotechnology) to develop recessive canker resistant grapefruit lines (Hongge et al., 2016).

This Research Topic demonstrates that grafting susceptible scions on resistant rootstocks is a valuable tool for the management of pests and diseases in vegetable crops and fruit trees, which effectively improves crop health and increases fruit yields. The continued development and utilization of resistant rootstocks also provide a rational alternative to the use of pesticides, which are harmful to the environment, for disease control.

## Author contributions

JT: Writing – original draft. DP: Writing – review & editing.
